# Characterization of Cell Surface Glycan Profiles in Human and Mouse Corneas Using Lectin Microarrays

**DOI:** 10.3390/cells12192356

**Published:** 2023-09-26

**Authors:** Rafael Martínez-Carrasco, Pablo Argüeso

**Affiliations:** Department of Ophthalmology, Tufts Medical Center, Tufts University School of Medicine, 150 Harrison Avenue, Boston, MA 02114, USA

**Keywords:** cornea, glycan, glycocalyx, lectin, microarray

## Abstract

The advent of high-throughput sequencing technologies has facilitated the profiling of glycosylation genes at a single-cell level in complex biological systems, but the significance of these gene signatures to the composition of the glycocalyx remains ambiguous. Here, we used lectin microarrays to characterize the composition of cell surface glycans in human and mouse corneas and determine its relationship to single-cell transcriptomic data. Our results identify a series of cell surface glycan signatures that are unique to the different cell types of the human cornea and that correlate, to a certain extent, with the transcriptional expression of glycosylation genes. These include pathways involved in the biosynthesis of O-glycans in epithelial cells and core fucose on stromal and endothelial cell surfaces. Moreover, we show that human and mouse corneas display some structural differences in terms of cell surface glycan composition. These results could provide insights into the specialized function of individual cell types in the cornea and foster the identification of novel cornea-specific biomarkers.

## 1. Introduction

The surface of the cell contains a meshwork of carbohydrate-enriched structures known as the glycocalyx. It is formed by the coordinated action of nucleotide sugar transporters, hydrolases, and a multitude of glycosyltransferases that synthesize and modify glycan chains as they transit through the endoplasmic reticulum and Golgi apparatus [1]. The resulting glycoconjugates are then inserted into the outer layer of the cell membrane, positioning their carbohydrate portions facing the extracellular space, where they can be further modified by extracellular enzymes. The glycocalyx exhibits variability in both composition and functions across different cell types and tissues, enabling it to fulfill specific roles based on the location of the cell within the body [2,3]. The glycocalyx is also dynamic, and its composition can change during cell growth, differentiation, and disease progression [4]. Identifying the composition of the glycocalyx is critical to the understanding of a range of cellular processes, including cell proliferation and differentiation, intercellular communication, interaction with the extracellular matrix, and the modulation of immune responses [4,5,6].

Advances in single-cell sequencing technologies are beginning to pave the way for predicting the glycosylation capacity of complex biological systems [5,7]. However, the significance of transcriptional signatures to the composition of the glycocalyx remains ambiguous. These single-cell sequencing approaches present limitations regarding the quantitation of lowly expressed genes and the results are highly sensitive to how clusters of cells are classified [7]. Moreover, mRNA levels do not always reflect enzyme levels directly [8], and these approaches are not intended to incorporate the contributions of the microenvironment to the shaping of the glycocalyx. Therefore, identifying the composition of the glycocalyx requires the integration of multiple experimental tools. Lectins are a family of proteins that possess the ability to bind carbohydrate structures [9]. This characteristic makes them highly valuable in deciphering the sugar codes present on cell surfaces [10]. Recent developments in lectin microarray technology enable rapid and sensitive analysis of complex glycans. These microarrays simplify glycomic profiling, offering high sensitivity and preserving the natural conformation of intact glycoproteins, since there is no need to release the glycans from the protein [11,12]. Lectin microarrays have been employed for multiple applications, including the discovery of stem cell biomarkers [13], discrimination between different cell types [14], disease detection [15], and the characterization of immune cell responses [16].

The cornea, serving as the outermost part of the eye, assumes a dual role as both a defensive barrier and the eye’s first dioptric element [17]. Anatomically, it is composed of three distinct anatomical layers: epithelium, stroma, and endothelium [18]. The limbus forms the transition zone between the peripheral cornea and the sclera and serves as the reservoir of adult stem cells responsible for replenishing the cornea. Previous studies have used lectins to define carbohydrate structures in the corneal layers of human and nonhuman species [19,20,21,22,23,24]. However, the scope of these experiments has been somehow limited by the reduced number of lectins tested and the reliance on fixatives known to cause artifacts and loss of antigenicity in histochemical assays [25]. In this study, we used lectin microarrays to perform a comprehensive analysis of the cell surface glycans present in the different compartments of the human cornea and examine their correlation with the transcriptional expression of glycosylation genes. Moreover, we used the lectin microarray to determine the structural character of the mouse cornea in terms of cell surface glycan composition.

## 2. Material and Methods

### 2.1. Cell Isolation from Human Corneas

Human postmortem corneoscleral tissues from 7 individuals of ages between 44 and 74 were provided by the Lions Eye Institute for Transplant and Research (Tampa, FL, USA) and Lions VisionGift (Portland, OR, USA). The iris, ciliary body, and excess conjunctiva were removed with the help of a scalpel. Portions of the corneal endothelium were then peeled off with jeweler forceps for production of primary cultures. The remaining corneal tissue was trephined with an 8 mm disposable biopsy punch (Integra Miltex, York, PA, USA) and separated from the limbal portion. The cornea and limbal portion were incubated individually for 2 h at 37 °C with 2.4 IU/mL Dispase II (Thermo Fisher Scientific, Waltham, MA, USA) in Dulbecco’s Modified Eagle Medium: Nutrient Mixture F-12 (DMEM/F12). The epithelia were scrapped with a scalpel, collected, and further digested with Gibco^TM^ TrypLE (Thermo Fisher Scientific) for 5 min. The resulting cell suspension was passed through a 70 µm cell strainer (Miltenyi Biotec; Bergisch Gladbach, Germany).

The corneal and limbal stroma were cut in small pieces, pooled, and placed in a MACS C tube (Miltenyi Biotec) containing 4 mg/mL collagenase A (Sigma-Aldrich, St. Louis, MO, USA) in DMEM. The stromal pieces were digested for 1 h at 37 °C using a gentleMACS dissociator (Miltenyi Biotec). After rinsing in DMEM, single cells were passed through a 70 µm cell strainer.

### 2.2. Culture of Human Corneal Endothelial Cells

The stripped corneal endothelium was placed in a culture plate coated with FNC Coating Mix^®^ (Athena Environmental Sciences, Baltimore, MD, USA). The pieces were left undisturbed overnight at 37 °C in a humidified 5% CO_2_ chamber in proliferative growth medium, composed of Gibco^TM^ Opti-MEM-I (Thermo Fisher Scientific) supplemented with 8% fetal bovine serum (FBS; Hyclone, Logan, UT, USA), 5 ng/mL human recombinant EGF (PeproTech, Rocky Hill, NJ, USA), 100 μg/mL bovine pituitary extract (Biomedical Technologies, Stoughton, MA, USA), 200 mg/L calcium chloride (Invitrogen, Carlsbad, CA, USA), 0.08% chondroitin sulfate (Sigma-Aldrich), 50 μg/mL gentamicin, and 1× antibiotic/antimycotic solution (Thermo Fisher Scientific). The following day, the culture medium was replaced, and the cells were fed every other day. To minimize endothelial to mesenchymal transition, a previously published dual media was employed [26]. Here, passage 1 cells were grown to confluency in the described proliferative growth medium and then switched to stabilization medium, composed of Opti-MEM-I supplemented with 4% fetal bovine serum, 50 μg/mL gentamicin, and 1× antibiotic/antimycotic solution. Cells were maintained in stabilization medium for 1 week. Then, the cells were collected for biotinylation and protein extraction or for RNA isolation.

### 2.3. Culture of Human Corneal Fibroblasts

Primary cultures of human corneal fibroblasts were grown following established protocols [27], wherein cells were allowed to adhere to the bottom of tissue culture plates using DMEM/F12 supplemented with 10% newborn calf serum (Thermo Fisher Scientific). Passage 1 cells were collected for biotinylation and protein extraction or for RNA isolation.

### 2.4. RNA Isolation and PCR

RNA was isolated using RNeasy Mini Kit (Qiagen, Valencia, CA, USA) following the manufacturer’s instructions. The samples were homogenized by 15 s of vortexing. Potential genomic DNA contamination was eliminated with DNase I (Invitrogen) prior to reverse transcription. First-strand cDNA was synthesized using the iScript cDNA synthesis kit (Bio-Rad, Hercules, CA, USA) following the manufacturer’s instructions.

The PCR reaction was carried out using iTaq Universal SYBR Green Supermix (Bio-Rad) with specific primers for *KRT15*, *KRT3*, *KERA*, *COL1A1*, and *NCAM1* (PrimeTime qPCR primers, Integrated DNA Technologies, Coralville, IA, USA). The following parameters were used: 30 s at 95 °C, followed by 30 cycles of 5 s at 95 °C and 30 s at 60 °C. The reaction products were separated by electrophoresis through 1% agarose gels in TAE buffer and visualized using a ChemiDoc MP Imaging System (Bio-Rad).

### 2.5. Mouse Corneal Tissue

Twelve-week-old C57BL/6J mice were acquired from the Jackson Laboratory (Bar Harbor, ME, USA) and used in accordance with institutional guidelines and the ARVO Statement for the Use of Animals in Ophthalmic and Vision Research. Mice were euthanized, and the cornea, including the limbus, was extracted by cutting with microscissors at the transitional zone with the conjunctiva. The iris was peeled off and the corneas were processed to obtain single cell suspensions as described above, using sequential incubation with Dispase II and TrypLE to isolate epithelial cells and digestion of the remaining tissue with collagenase A. Single cell suspensions from whole corneas were pooled and subjected to cell surface protein biotinylation.

### 2.6. Cell Surface Protein Biotinylation

Cell surface proteins from human and mouse cell suspensions were biotinylated using EZ-Link NHS-Biotin (Thermo Fisher Scientific) according to the manufacturer’s instructions. Briefly, cells were incubated 10 min at room temperature in 0.25 mg/mL NHS-biotin solution in PBS. Then, the cells were washed in TBS to quench and remove all excess reagent and lysed in Triton X-100 buffer (1% Triton X-100, 50 mM Tris base pH 8.0, 150 mM NaCl) containing 1 mM PMSF and a protease inhibitor cocktail (Roche Applied Science, Penzberg, Germany) for 1 h at 4 °C. The insoluble material was cleared by centrifugation at 15,000× *g* for 30 min at 4 °C.

### 2.7. Lectin Microarray

Cell surface glycans were assessed using a lectin microarray following the manufacturer’s instructions (GA-Lectin-70; RayBiotech Life, Peachtree Corners, GA, USA). The location and glycan binding specificity of each lectin in the microarray (70 different lectins displayed in duplicate) is summarized in Supplementary Table S1. Samples were pooled to mitigate inter-individual variability. Biotinylated cell lysates pooled from 3 to 4 individuals were used for the analysis of corneal, limbal, stromal and endothelial samples, whereas lysates from 3 additional individuals were pooled for fibroblast analysis. Biotinylated cell lysates from 14 animals were pooled for the analysis of the whole mouse cornea. Lysates were dialyzed overnight at 4 °C in PBS with three buffer changes. The microarray slides were incubated with 500 ng of dialyzed protein overnight at 4 °C. The slides were washed under gentle shaking and labeled with Cy3-streptavidin for 1 h at room temperature. The slides were subsequently washed, dried, and scanned by RayBiotech. The background fluorescence was subtracted from the fluorescence intensity, which was then averaged for each duplicate lectin spot.

### 2.8. Single-Nucleus RNA Sequencing (snRNAseq) Data Analysis

The cell atlas of the human ocular anterior segment has been recently reported and the datasets deposited at the Gene Expression Omnibus repository (GSE199013) [28]. The processed read count matrix of the human cornea and limbus were imported into R (Version 4.3.1) and converted to a Seurat object using the Seurat R package (Version 4.3.0.1). The data were filtered to remove cells with more than 30,000 reads, less than 1000 genes, or more than 10% mitochondrial reads. Gene expression normalization was applied to each dataset using the *LogNormalize* method, and the top 2000 variable genes were selected using *FindVariableFeatures*. A principal component analysis was run on the variable genes, followed by the *FindNeighbors* and *FindClusters* functions using the Louvain algorithm to generate cell clusters with 50 principal components (PC) and a resolution of 0.6. Clusters were then regrouped based on specific markers. Cells expressing *KRT5* in the limbal and corneal datasets were clustered as limbal and corneal epithelia, respectively. Cells expressing *CDH19* but lacking *ACTA2* and *MLANA* in both datasets were combined and clustered as endothelial cells. The remaining cells in both datasets, excluding the *MLANA* positive cells, were grouped in a new cluster as stromal cells. The *FindAllMarkers* function was used to identify differentially expressed genes between the clusters. 

### 2.9. Statistical Analysis

Principal component analysis of lectin binding was performed using Prism 9.0 (GraphPad Software, San Diego, CA, USA) for Mac OS X. The data were first quantile-normalized to remove systematic biases or effects due to the distribution of the variables. Lectins with intensity values lower than the negative control in all samples were removed from the analysis. Then, principal component analysis was performed to reduce the dimensionality of the data. The first 3 principal components were selected, which explained at least 75% of the variance in the data. The loading values of each lectin were calculated for PC1, PC2, and PC3. The coefficient of variation, defined as the ratio of the standard deviation to the mean, was calculated for each lectin using Excel (V 16.74), establishing a cutoff value of 30%.

## 3. Results and Discussion

**Figure 1:**
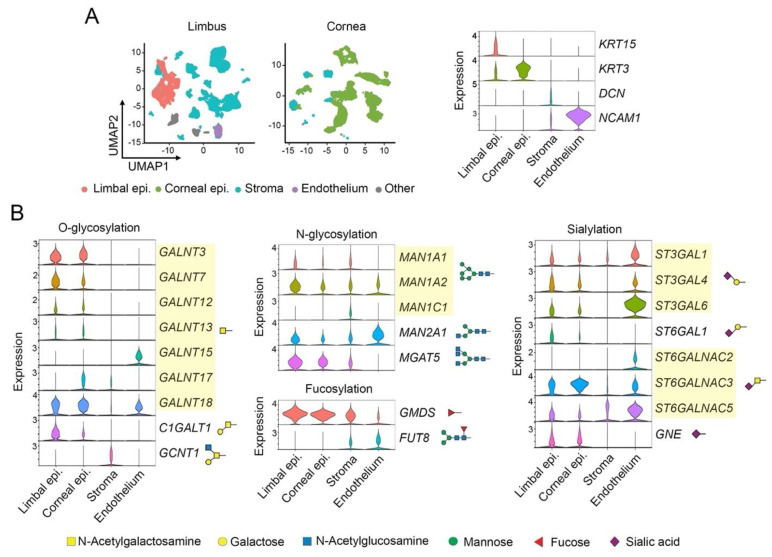
Single-nucleus RNA sequencing (snRNAseq) data analysis. (**A**) Integrated UMAP showing the clustering of different cell types in the human limbus and cornea. The violin plots display key markers for epithelial cells in the limbus and cornea, stromal cells, and endothelial cells. (**B**) Violin plots showing differentially expressed genes involved in the biosynthesis of O-glycans, N-glycans, and derivatives containing fucose and sialic acid. The symbols indicate the glycan structures synthesized by each enzyme.

The advancement in single-cell RNA sequencing offers an extraordinary opportunity to explore the glycosylation of individual cells in complex tissues like never before. In our study, we used a public snRNAseq dataset containing transcriptomes from 51,306 and 37,485 single nuclei to investigate the expression of glycosylation genes in the human limbus and cornea, respectively [28]. Using established markers, we selected four clusters of cells populating the distinct layers of the anterior segment: limbal epithelium, corneal epithelium, stromal cells, and endothelial cells (Figure 1A). Next, we performed a differential expression analysis to identify glycosylation genes that show significant changes in expression between the four different groups of cells (Figure 1B). We found that, compared to stromal and endothelial cells, the limbal and corneal epithelia express higher levels of polypeptide GalNAc transferases (*GALNT3*, *7*, *12*, *13*, *17*, *18*) and *C1GALT1*, the only enzyme responsible for the biosynthesis of core 1 O-glycans [29]. These results were expected, since the limbal and corneal epithelia are known to synthesize significant amounts of membrane-bound mucins carrying core 1 O-glycans, particularly in the most superficial layers of the epithelia [30,31,32].

Examination of additional glycosylation pathways revealed that epithelial cells express high levels of *GMDS*, while stromal and endothelial cells express *FUT8* (Figure 1B). GMDS and FUT8 facilitate fucose metabolism through the de novo pathway and transfer fucose to the innermost N-acetylglucosamine residue of N-glycans, a process called core fucosylation [33]. The distinctive expression of these genes suggests that fucosylation is differentially induced among corneal compartments and that the linkage of fucose to the glycan chain varies according to cell type, with stromal and endothelial cells displaying core fucose more abundantly. Additionally, the analysis of expression data reveals low transcription of certain sialyltransferases in stromal cells and the preferential expression of *MGAT5*, one of the most important enzymes for the biosynthesis of N-glycans that are elongated with poly N-acetyllactosamine to create ligands for the galectin family of mammalian lectins, in epithelial cells [34]. The presence of this enzyme and galectin-3 in the cornea have been shown to play a critical role in modulating neovascularization in a clinically relevant corneal suture model [35].

**Figure 2:**
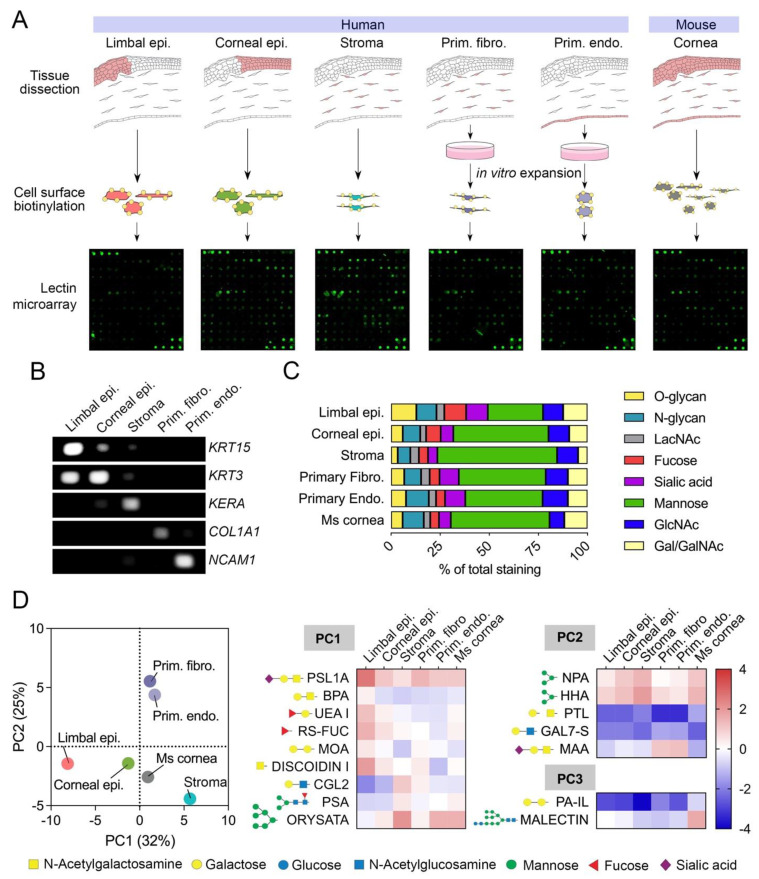
Cell surface glycan profiles of human and mouse corneas as determined by lectin microarray. (**A**) Schematic representation of the workflow used for lectin microarray analysis. (**B**) PCR detection of cell type-specific markers in RNA extracted from limbal epithelium, corneal epithelium, stroma, primary fibroblasts, and primary endothelial cells. (**C**) Relative staining of different groups of lectins classified according to carbohydrate specificity (from Supplementary Table S1). The fluorescence intensity of each lectin in the microarray was normalized within each sample and expressed as percentage of total intensity. (**D**) Score plot for principal component 1 (PC1) and 2 (PC2) from the principal component analysis of 65 lectins showing positive fluorescence intensity after quantile normalization. The heatmap shows log2 normalized intensity values from lectins with a coefficient of variation higher than 30% and loading values for PC1, PC2, and PC3 > 0.80 or <−0.80. The symbols indicate the glycan structures recognized by each lectin.

Although the analysis of the snRNAseq dataset was important to define the glycosylation transcriptome of the limbus and cornea at a single cell level, the significance of these results to the composition of the glycocalyx remained unclear. Toward this purpose, we analyzed biotinylated cell surface proteins in healthy human corneas by lectin microarray (Figure 2A). Here, the different compartments of the cornea were anatomically separated into limbal epithelium, corneal epithelium, stroma, and endothelium to allow comparison with the snRNAseq dataset. In order to obtain an adequate amount of protein for the assay, human corneal fibroblasts and endothelial cells were cultured and expanded in vitro. The identity of the samples was confirmed by PCR, using specific markers for the different regions of the cornea (Figure 2B). Limbal epithelial cells were characterized by *KRT15* expression, whereas corneal epithelial cells and stromal cells expressed *KRT3* and *KERA*, respectively [36]. Furthermore, the cultures of primary fibroblasts expressed *COL1A1*, whereas the primary corneal endothelial cells expressed *NCAM1* but not *COL1A1*, implying the absence of a fibroblastic phenotype in these cells.

The use of a lectin microarray allowed us to assess the presence of carbohydrate structures on specific corneal cell types based on lectin specificity (Figure 2C). We found that mannose-binding lectins bound to stromal cells more frequently than to limbal epithelial cells, suggesting a greater prevalence of high-mannose N-glycans on the glycocalyx of stromal cells. In contrast, epithelial tissues displayed higher intensities for lectins recognizing O-glycans and sialic acid in comparison to the stroma, as expected from the transcriptional data and known expression of membrane-bound mucins carrying sialylated core 1 O-glycans at the ocular surface epithelia [30,31,32]. Other carbohydrate structures appeared to have similar distributions across the different compartments of the cornea, primarily those containing N-acetyllactosamine and N-acetylglucosamine.

To further investigate cell surface glycan profiles in the cornea and their relationship to snRNAseq data, we performed principal component analysis on normalized lectin microarray intensity values (Figure 2D, Supplementary Table S2). The first three principal components (PC1, PC2, and PC3) accounted for a substantial proportion of the variance in the data, with PC1 explaining 32%, PC2 explaining 25%, and PC3 explaining 18% of the total variance. PC1 and PC2 separated the human samples into epithelial, stromal, and cultured cells, suggesting the presence of glycan motifs in the glycocalyx that differentiate these three groups of cell types. We then selected those lectins with a coefficient of variation higher than 30% and loading values for PC1, PC2, and PC3 > 0.80 or <−0.80 for further analyses. The PC1 loadings indicated that α2-6 sialic acid (PSL1A), O-glycans (BPA, DISCOIDIN I), and fucose (UEA I, RS-FUC) were predominantly present in the limbal epithelial compartment. Conversely, binding of ORYSATA to high mannose residues led to marked positive loadings in PC1, suggesting a relatively higher abundance of these glycans in stromal and endothelial cells. While the snRNAseq data correlated with the abundance of core 1 O-glycans and fucose, but not core fucose (as shown by PSA binding), in epithelial cells, the connection of high mannose residues with specific gene expression in stromal and endothelial cells was more ambiguous.

Analysis of the PC2 loading highlighted the abundance of α2-3 sialic acid (MAA) in primary cultures of fibroblasts and endothelial cells (Figure 2D), the latter correlating with a marked expression of α2-3 syalyltransferases by snRNAseq (Figure 1B) and a previous immunofluorescence study showing MAA staining exclusively in the corneal endothelium [37]. The PC3 loading provided further information on how the human cornea compares to its mouse counterpart. PA-IL, a lectin with affinity toward α-galactosylated glycans [38], bound weakly to human tissue compared to mouse, as it did Malectin, which revealed a more abundant presence of glucosylated high-mannose N-glycans in the mouse cornea.

The use of lectin microarrays in this study presents several limitations. One is the efficiency of the biotinylation reaction, which can be influenced by the intrinsic conformation and cell surface distribution of the target proteins. Also, the presence of endogenous lectins in our samples could impair the recognition of certain carbohydrate epitopes in the microarray by steric hindrance. We believe this is the case with galectin-3, one of the most highly expressed glycogenes at the human ocular surface [39]. Surprisingly, we did not detect binding of galectin-3 in the array to any of the biotinylated samples tested in our study (Supplementary Table S2). Yet, multiple lines of evidence indicate that several carbohydrate epitopes recognized by galectin-3 are present in the cornea, particularly in membranous regions of the corneal epithelium [40,41,42,43]. We hypothesize that endogenous galectin-3 binds cell surface counterreceptors present in corneal cells and interferes with galectin-3 recognition in the lectin microarray. Such a scenario might result in an underestimated count of the number of available cell surface glycans, which is a limitation that should be considered in future studies.

## 4. Conclusions

This study identifies a series of cell surface glycan signatures that are unique to the different cell types of the human cornea. These include pathways involved in the biosynthesis of O-glycans in epithelial cells and core fucose on stromal and endothelial cell surfaces. Moreover, we show a certain degree of correlation between the composition of the glycocalyx and the transcriptional expression of glycosylation genes in the human cornea. The data also provide evidence indicating that the human and mouse corneas display some structural differences in terms of cell surface glycan composition. We believe that these findings could provide valuable insights into the specialized function of individual cell types in the cornea, facilitate the discovery of biomarkers that are specific to the cornea, and could have implications for understanding the ocular phenotype in pathologies where glycosylation has been shown to be altered [22,44].

## Data Availability

The data presented in this study are available in the article.

## Supplementary Materials

The following supporting information can be downloaded at: https://www.mdpi.com/article/10.3390/cells12192356/s1.
