# Supplementary material for: Characterization of Cell Surface Glycan Profiles in Human and Mouse Corneas Using Lectin Microarrays
Source: Cells. 2023 Sep 26;12(19):2356. doi: 10.3390/cells12192356 (PMC10572028; doi:10.3390/cells12192356)
Supplement: Supplementary file 1 [file cells-12-02356-s001.zip › cells-2570195-supplementary.pdf]

Supplementary Table S1. Position and glycan binding specificity of lectins in the microarray.

Symbol	*Lectin*	*Position*	*Glycan specificity*
O-Glycan			
ACL	*Amaranthus caudatus* lectin	2CD	Galβ3GalNAc
BPA	*Bauhinia purpurea* agglutinin	3AB	Galβ3GalNAc
DISCOIDIN I	*Dictyostelium discoideum I*	4AB	αGalNAc
GS-I	*Griffonia simplicifolia I*	6GH	αGal, α3GalNAc
Jacalin; AIA	Jacalin (*Artocarpus intergrifolia agglutinin)*	7AB	Galβ3GalNAc
MPL	*Maclura pomifera* lectin	8IJ	Galβ3GalNAc
PNA	Peanut agglutinin	10CD	Galβ3GalNAc
SJA	*Sophora japonica* agglutinin	11GH	βGalNAc
SNA-II	*Sambucus nigra* agglutinin II	11KL	GalNAc > Gal
VVA	*Vicia villosa* agglutinin	12KL	GalNAc
N-Glycan			
LcH A	*Lens Culinaris* hemagglutinin	7EF	αMan, αGlc, core with Fucα1-6
MALECTIN	Human malectin	8EF	Glc2-N-biose
PHA-E	*Phaseolus vulgaris* Erythroagglutinin	9IJ	Galβ4GlcNAcβ2Manα6(GlcNAcβ4)(GlcNAcβ4Manα3)Manβ4
PHA-L	*Phaseolus vulgaris* Leucoagglutinin	9KL	Galβ4GlcNAcβ6(GlcNAcβ2Manα3)Manα3
PHA-P	*Phaseolus vulgaris* Phytohemagglutinin	10AB	Triantenary, tetraantenary structures
PSA	*Pisum sativum* agglutinin	10GH	αMan, αGlc, core with Fucα1-6
N-Acetyllactosamine			
CGL2	*Coprinopsis cinerea* lectin 2	3EF	βGal
CNL	*Clitocybe nebularis* lectin	3GH	α/βGalNAc, GalNAcβ1-4GlcNAc
ECA	*Erythrina crista-galli* agglutinin	4GH	Galβ4GlcNAc
GAL1	Human galectin1	5AB	Branched LacNAc, Gal
GAL1-S	Human galectin1-S	5CD	Branched LacNAc
GAL2	Human galectin2	5EF	Branched LacNAc

GAL3	Human galectin3	5GH	poly LacNAc
GAL3C-S	Human galectin3C-S	5IJ	poly LacNAc
GAL7-S	Human galectin7-S	5KL	Galβ1-3GlcNAc
GAL9	Human galectin9	6AB	poly LacNAc
LSL-N	*Laetiporus sulphureus* lectin	8AB	LacNAc
Fucose			
AAA	*Anguilla anguilla* agglutinin	1IJ	αFuc
AAL	*Aleuria aurantia* lectin	1KL	αFuc
BC2LCN	*Burkholderia cenocepacia* lectin CN	2KL	Fucα1-2Galβ1-3GalNAc/GlcNAc
LTL	*Lotus tetragonolobus* lectin	7KL	α1-3Fuc (Lex)
PA-IIL	*Pseudomonas aeruginosa* lectin II	9CD	αFuc, αMan
RS-FUC	*Ralstonia solanacearum*	11AB	αFuc
UEA I	*Ulex europaeus* agglutinin I	12EF	α1-2Fuc
Sialic acid			
ACG	*Agrocybe cylindracea* galectin	2AB	α2-3 Sialic Acid
MAA	*Maackia amurensis* agglutinin I	8CD	Neu5Acα2-3Gal(β1–4)GlcNAc/Glc
PSL1A	*Polyporus squamosus* lectin 1A	10IJ	α2-6 Sialic Acid
SAMB	*Sambucus sieboldiana*	11CD	NeuAcα2-6Gal, GalNAc
SNA-I	*Sambucus nigra* aagglutinin I	11IJ	Neu5Acα2-6Gal, GalNAc
Mannose			
ASA	*Allium sativum* agglutinin	2EF	αMan
BANLEC	*Musa acuminata* (banana lectin)	2GH	αMan, αGlc
BC2L-A	*Burkholderia cenocepacia* lectin A	2IJ	High-mannose
CALSEPA	*Calystegia sepium* agglutinin	3CD	High-mannose
Con A	Concanavalin A	3IJ	αMan, αGlc
GNA	*Galanthus nivalis* agglutinin	6CD	αMan
GRFT	*Griffithia sp.*	6EF	High-mannose
HHA	*Hippeastrum hybrid* agglutinin	6KL	αMan
LENTIL	Lentil	7IJ	αMan, αGlc

NPA	*Narcissus pseudonarcissus* agglutinin	8KL	αMan
ORYSATA	*Oryza sative* agglutinin	9AB	High-mannose
PALa	*Phlebodium aureum* lectin	9GH	High-mannose
VFA	*Vicia faba* agglutinin	12IJ	αMan
N-Acetylglucosamine			
DSA	*Datura stramonium* agglutinin	4EF	GlcNAc
F17AG	*Escherichia coli*	4KL	GlcNAc
GS-II	*Griffonia simplicifolia* II	6IJ	α/βGlcNAc
LEA	*Lycopersicon esculentum* agglutinin	7GH	GlcNAc
STL	*Solanum tuberosum* lectin	12AB	GlcNAc
UDA	*Urtica dioica* agglutinin	12GH	Poly β(1,4)GlcNAc
UEA-II	*Ulex europaeus* agglutinin II	12CD	GlcNAc
WGA	Wheat germ agglutinin	13CD	GlcNAc, Sialic acid
Galactose and N-acetylgalactosamine			
DBA	*Dolichos biflorus* agglutinin	3KL	αGalNAc
DISCOIDIN II	*Dictyostelium discoideum II*	4CD	Gal, LacNAc
EEL	*Eunonymus europaeus* lectin	4IJ	Galα3Gal
LBA	*Phaseolus lunatus* agglutinin	7CD	GalNAcα(1,3)[αFuc(1,2)]Gal
MOA	*Marasmius oreades* agglutinin	8GH	Galα1-3Galβ1-4GlcNAc, Galα1-3Gal
PA-IL	*Pseudomonas aeruginosa* lectin I	9EF	Galα1-3(4)Gal
PPL	*Pleurocybella porrigens* lectin	10EF	α/βGalNAc
PTL	*Psophocarpus tetragonolobus* lectin	10KL	GalNAc, Gal
SBA	Soybean agglutinin	11EF	α > βGalNAc
WFA	*Wisteria floribunda* agglutinin	13AB	GalNAc

Supplementary Table S2. Quantile-normalized fluorescence intensity of lectin binding to cell surface proteins isolated from human and mouse corneas. The coefficient of variation (CV) was calculated to measure the dispersion of the quantile-normalized intensity. Principal component analysis (PCA) was used to reduce the dimensionality of the datasets. Data in red denotates values with a CV higher than 30% and loading values for PC1, PC2 and PC3 > 0.80 or < -0.80. Schematic of the structures recognized by each lectin are shown.

		Normalized intensity							Loading values		
	Lectin	Limbal epi.	Central epi.	Stroma	Prim. fibro.	Prim. endo.	Ms cornea	CV (%)	PC1	PC2	PC3
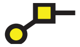	ACL	0.50	0.29	0.26	0.36	1.32	0.29	80.95	-0.16	0.62	-0.01
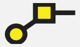	BPA	1.24	0.68	0.59	0.65	0.68	0.77	30.84	-0.93	-0.15	0.04
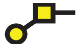	DISCOIDIN I	3.80	1.37	1.00	1.32	0.50	1.04	77.58	-0.80	-0.30	-0.26
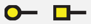	GS-I	0.29	0.22	0.31	0.36	0.29	0.30	14.64	0.27	0.31	-0.09
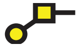	Jacalin; AIA	1.10	0.72	0.44	0.77	0.65	0.87	28.99	-0.88	0.16	0.40
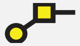	MPL	0.23	0.23	0.15	0.17	0.21	0.14	22.33	-0.67	0.25	-0.34
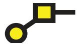	PNA	0.80	0.63	0.63	0.59	0.55	0.89	19.37	-0.45	-0.58	0.53
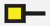	SJA	0.17	0.15	0.18	0.02	0.18	0.17	43.92	-0.09	-0.67	0.13
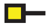	SNA-II	0.11	0.11	0.12	0.12	0.11	0.11	5.26	0.58	0.11	-0.54
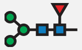	LcH A	0.53	0.77	0.77	0.44	2.66	0.55	88.92	0.29	0.29	-0.02
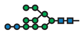	MALECTIN	1.00	0.87	0.57	0.53	0.63	3.24	91.92	-0.22	-0.44	0.86
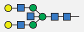	PHA-E	0.74	1.04	0.93	2.33	1.04	0.80	51.57	0.30	0.74	-0.20
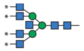	PHA-L	0.57	0.74	0.29	0.63	0.89	0.65	31.95	-0.36	0.62	0.53
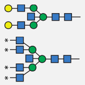	PHA-P	3.47	2.66	1.24	0.93	0.87	1.32	60.87	-0.79	-0.52	-0.16
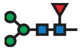	PSA	0.63	0.65	1.37	0.89	1.00	1.24	31.49	0.84	-0.20	0.22

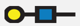	CGL2	0.27	0.44	2.07	1.24	0.77	0.74	70.86	0.93	0.05	-0.19
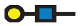	CNL	0.10	0.08	0.11	0.14	0.12	0.10	17.08	0.33	0.66	-0.15
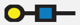	ECA	0.26	0.30	0.27	0.29	0.29	0.33	8.71	0.27	0.04	0.90
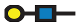	GAL1	0.05	0.04	0.08	0.08	0.05	0.06	31.89	0.55	0.05	-0.14
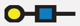	GAL1-S	N/D	N/D	N/D	N/D	N/D	N/D				
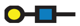	GAL2	0.19	0.17	0.17	0.18	0.23	0.25	17.60	0.02	0.16	0.81
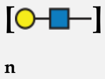	GAL3	N/D	N/D	N/D	N/D	N/D	N/D				
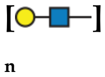	GAL3C-S	0.15	0.19	0.50	0.20	0.19	0.53	59.42	0.67	-0.58	0.33
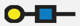	GAL7-S	0.25	0.25	0.20	0.38	0.36	0.18	30.89	-0.08	0.95	-0.28
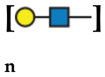	GAL9	N/D	N/D	N/D	N/D	N/D	N/D				
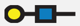	LSL-N	0.33	0.20	0.53	0.23	0.26	0.19	43.99	0.22	-0.37	-0.77
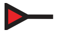	AAA	0.12	0.29	0.19	0.15	0.14	0.15	36.63	0.26	-0.33	-0.10
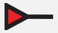	AAL	2.15	1.81	1.04	0.87	0.59	1.00	48.30	-0.71	-0.54	-0.22
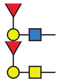	BC2LCN	0.91	0.53	0.65	0.29	0.20	0.20	61.83	-0.44	-0.52	-0.72
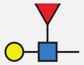	LTL	0.06	0.06	0.06	0.11	0.06	0.08	27.41	0.26	0.53	0.28
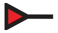	PA-IIL	N/D	N/D	N/D	N/D	N/D	N/D				
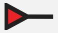	RS-FUC	2.86	1.51	1.10	1.00	0.74	1.20	54.11	-0.84	-0.47	-0.18
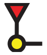	UEA I	2.33	1.24	0.89	0.74	0.72	1.10	51.59	-0.87	-0.49	-0.06

α2-3	ACG	0.36	0.36	0.36	0.57	0.53	0.29	27.11	0.19	0.90	-0.39
α2-3	MAA	0.59	0.80	0.72	2.15	2.33	0.41	72.30	0.26	0.90	-0.30
α2-6	PSL1A	6.29	2.15	1.51	2.66	2.07	2.07	62.74	-0.94	0.11	-0.14
α2-6	SAMB	0.68	0.41	0.48	0.48	0.41	0.44	21.06	-0.71	-0.20	-0.38
α2-6	SNA-I	1.51	1.00	0.80	0.80	0.80	0.93	28.37	-0.94	-0.33	-0.06
	ASA	1.61	3.47	5.23	3.24	2.15	2.33	43.08	0.77	-0.26	-0.38
	BANLEC	2.66	5.23	3.47	5.23	3.47	5.23	27.35	0.37	0.17	0.54
	BC2L-A	0.48	0.50	0.74	0.68	0.57	0.36	25.05	0.49	0.28	-0.82
	CALSEPA	0.72	1.61	1.20	1.81	1.81	0.57	42.44	0.37	0.63	-0.46
	Con A	3.24	4.22	3.24	3.80	3.24	3.80	11.46	0.00	0.04	0.44
	GNA	5.23	6.29	6.29	6.29	6.29	6.29	7.04	0.87	0.18	0.28
	GRFT	1.04	3.80	2.15	4.22	3.80	3.47	39.78	0.59	0.48	0.44
	HHL	1.81	2.33	3.80	1.61	1.37	2.66	39.41	0.43	-0.87	-0.06
	LENTIL	0.65	1.20	1.32	1.37	1.20	1.37	23.03	0.88	0.15	0.32
	NPA	1.32	2.07	2.66	1.04	1.24	2.15	36.62	0.41	-0.88	0.10
	ORYSATA	0.87	1.32	4.22	1.20	2.86	2.86	58.71	0.83	-0.30	0.16
	PALa	0.55	0.59	1.61	0.72	1.61	1.51	48.01	0.76	-0.12	0.28
	VFA	0.08	0.10	0.14	0.06	0.06	0.06	36.59	0.19	-0.67	-0.66

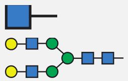	DSA	1.20	1.10	2.86	1.51	1.51	1.61	38.96	0.78	-0.33	-0.27
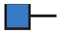	F17AG	0.91	3.24	0.68	2.07	1.10	0.68	70.33	-0.16	0.44	-0.13
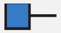	GS-II	0.77	0.55	0.38	0.55	0.48	0.59	23.42	-0.94	0.07	0.27
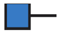	LEA	0.21	0.31	0.33	0.19	0.22	0.27	22.82	0.43	-0.78	0.00
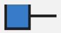	STL	2.07	0.89	2.33	2.86	5.23	1.81	58.33	0.27	0.59	-0.12
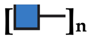	UDA	0.30	0.48	0.87	0.26	0.27	0.22	61.76	0.40	-0.58	-0.67
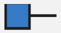	UEA-II	0.41	0.27	0.29	0.30	0.30	0.31	15.68	-0.75	-0.07	-0.11
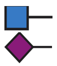	WGA	0.22	0.21	0.22	0.22	0.25	0.26	8.23	0.22	0.11	0.72
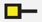	DBA	4.22	2.86	1.81	3.47	4.22	4.22	28.35	-0.59	0.48	0.59
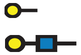	DISCOIDIN II	0.38	0.38	0.30	0.50	0.44	0.48	17.53	-0.12	0.71	0.65
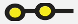	EEL	0.36	0.36	0.41	0.27	0.36	0.50	19.93	0.14	-0.75	0.52
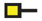	LBA	0.29	0.57	0.23	0.41	0.33	0.36	32.35	-0.17	0.33	0.36
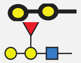	MOA	1.37	0.93	0.55	1.10	0.93	0.72	30.61	-0.84	0.53	-0.10
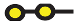	PA-IL	0.14	0.12	0.06	0.25	0.15	0.63	92.51	-0.13	0.22	0.90
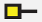	PPL	0.20	0.14	0.10	0.21	0.17	0.12	27.20	-0.60	0.76	-0.14
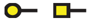	PTL	0.18	0.18	0.25	0.10	0.10	0.38	52.77	0.09	-0.87	0.44
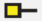	SBA	0.44	0.33	0.21	0.31	0.31	0.21	28.88	-0.83	0.39	-0.34
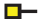	WFA	0.31	0.26	0.36	0.33	0.38	0.23	18.39	0.24	0.43	-0.72

N/D: Not detected. VVA binding was saturated and excluded from the analysis.
